# Patient-specific CFD simulation of intraventricular haemodynamics based on 3D ultrasound imaging

**DOI:** 10.1186/s12938-016-0231-9

**Published:** 2016-09-09

**Authors:** A. M. Bavo, A. M. Pouch, J. Degroote, J. Vierendeels, J. H. Gorman, R. C. Gorman, P. Segers

**Affiliations:** 1 ELIS Department, IBiTech-bioMMeda, Ghent University, Ghent, Belgium; 2 Gorman Cardiovascular Research Group, University of Pennsylvania, Philadelphia, PA USA; 3 Department of Flow, Heat and Combustion Mechanics, Ghent University, Ghent, Belgium

**Keywords:** CFD model with prescribed moving boundaries, Real-time transesophageal ultrasound images, Patient-specific modeling, Intraventricular flow

## Abstract

**Background:**

The goal of this paper is to present a computational fluid dynamic (CFD) model with moving boundaries to study the intraventricular flows in a patient-specific framework. Starting from the segmentation of real-time transesophageal echocardiographic images, a CFD model including the complete left ventricle and the moving 3D mitral valve was realized. Their motion, known as a function of time from the segmented ultrasound images, was imposed as a boundary condition in an Arbitrary Lagrangian–Eulerian framework.

**Results:**

The model allowed for a realistic description of the displacement of the structures of interest and for an effective analysis of the intraventricular flows throughout the cardiac cycle. The model provides detailed intraventricular flow features, and highlights the importance of the 3D valve apparatus for the vortex dynamics and apical flow.

**Conclusions:**

The proposed method could describe the haemodynamics of the left ventricle during the cardiac cycle. The methodology might therefore be of particular importance in patient treatment planning to assess the impact of mitral valve treatment on intraventricular flow dynamics.

**Electronic supplementary material:**

The online version of this article (doi:10.1186/s12938-016-0231-9) contains supplementary material, which is available to authorized users.

## Background

With the increasing incidence of heart failure, there is a renewed clinical interest in ventricular vortex dynamics that are thought to contribute to the energetically efficient filling and emptying of the heart. Computational models based on patient specific data, can contribute to our understanding of ventricular pathophysiology and the efficiency of ventricular dynamics. Based on the currently available imaging techniques of the cardiac structures, it is possible to generate advanced computational models which can, within the limits of the chosen numerical tool, reproduce a realistic scenario and provide additional information on the fluid-dynamics of the left ventricle (LV) [1].

As the human heart is a highly motile structure, its motion has to be included in any numerical simulation which studies the intraventricular flows. More specifically, this can be done via multi-physics simulations (fluid–structure interaction simulations and electromechanical models) or with a computational fluid dynamic (CFD) simulation with moving boundaries. For the purposes of this paper, only the latter will be described. In basic CFD models with moving boundaries, the geometry of the LV is simplified and consists in a prolated ellipsoid. Its kinematics can be prescribed by one parametric equation, which regulates the contraction and dilatation of the structure [2–6]. In more advanced models, if the geometry is realistic and/or patient-specific, the motion of the ventricular walls has to be based on a priori knowledge of their position, information that is normally derived from clinical images [7–13].

When studying the intraventricular flows, especially during diastole, the influence of the cardiac valves is critical [9]. The presence of the valves in such a model has been considered to be one of the crucial details of the problem [2, 9–14]. Due to its complex structure, however, the inclusion of the full mitral valve (MV) in a numerical simulation is not straightforward. Furthermore, the inclusion of a patient-specific mitral valve can be limited by the spatial resolution of the available imaging technique. Especially in the case of magnetic resonance images (MRI), the resolution is not adequate (yet) to obtain the 3D geometry of the valvular leaflets [8, 11, 12]. In several models, the 3D valve is omitted and to overcome the absence of the 3D valve, different approaches have been reported in the past years. The simplest model of the valve that can be used is the on–off approach [6, 8, 10]. With this technique, the switch between the open and closed configuration (and vice versa) occurs instantaneously, with no intermediate position. As a second option, the valve is represented with a time-varying planar orifice. This can be done by imposing a gradual aperture of the valvular orifice [15] or can be obtained from MRI images by projecting the valvular orifice on a plane [11, 12]. However, in this case, only the influence of the planar time-varying orifice is taken into account, with no spatial details of the 3D geometry of the annulus and, most importantly, of the 3D motion of the MV leaflets. In a few cases, the complete structure of the mitral valve is included in the model [7, 9, 13, 16]. In [9, 13, 16], the valve morphology is generated on the basis of a database of healthy subjects, resulting in a simplified shell structure. Its motion is prescribed by means of physiological angles of the leaflets, derived from echocardiographic measurements and physiology atlases. In [7], on the contrary, the availability of high-resolution CT (computed tomography) images allowed for the generation of a valvular geometry based on landmark positioning and a statistical model. The CFD simulations with moving walls for the ventricle alone could be performed with the ALE (Arbitrary Lagrangian–Eulerian) mesh discretization [e.g. 10–12]. On the contrary, the inclusion of the moving mitral valve was treated with an Immersed Boundary (IB) approach for the mesh discretization, resulting in either a full IB simulation [6] or in a combined IB-ALE simulation (ALE for the LV and IB for the MV) [12].

The goal of this work is to generate a CFD-based model with moving boundaries using the ALE formulation for the entire domain, to study the intraventricular flow in a 3D patient-specific framework. The model is based on segmented real-time transesophageal echocardiographic images (rt-TEE), from which the patient-specific geometry and the position of the LV and fully three-dimensional MV are derived. Compared to the available literature [e.g. 5, 6, 9, 11, 12], the proposed model and framework has the advantage of being patient-specific in both the LV and MV geometry and kinematics and the model of the valve is fully three-dimensional, not requiring additional assumptions over its geometry and thickness. The presence of the valve provides more realistic flow patterns during diastole, while during systole the coaptation is provided with a dedicated contact function.

## Methods

### Patient selection and ultrasound images segmentation

**Fig. 1:**
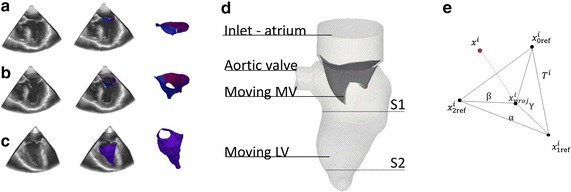
From rt-TEE ultrasound images to segmented triangulated surfaces. **a** MV in closed configuration. **b** MV in open configuration. **c** LV during systole

For this pilot study, one patient was selected on the basis of the clinical information and images available. The selection criteria for the patient were the inclusion of the entire MV and LV structures in the field of view of the pre-operative rt-TEE images and the absence of stitching artifacts. From the clinical point of view, the absence of mitral regurgitation was required. The imaging protocol was approved by the University of Pennsylvania School of Medicine Institutional Review Board. Major clinical features included severe aortic stenosis, moderate global systolic dysfunction and severe hypokinesis of the LV, the patient was identified for aortic valve replacement. Pre-operative rt-3D TEE gated images were acquired with an iE33 scanner (Philips Medical Systems, Andover, MA). The frame rate was 17–30 Hz with an image depth of 14–17 cm. The images were acquired over four consecutive cardiac cycles and reconstructed into one cardiac cycle to obtain the desired full field of view. The rt-3DTEE images contains the entire LV and MV in the field of view. The segmentation of the MV in these images was performed with a combination of multi-atlas joint label fusion and deformable medial modeling techniques, which generate 3D geometric models of the MV [17]. The model accurately replicates different geometrical configurations (closed in Fig. 1a, and open in Fig. 1b) of the valve and captures the atrial and ventricular surfaces with a high level of detail. Furthermore, the technique represents the valve volumetrically, which enables localized measurement of leaflet thickness. To obtain similar image-derived models of the LV without reference data for multi-atlas label fusion, semi-automated image segmentation of the LV was performed in ITK-SNAP [18] at one frame in systole (Fig. 1c), and deformable registration [19] was used to propagate that segmentation to all the other frames in the image series. Further details on the segmentation technique are beyond the scope of this paper, and the reader is referred to [17] for more information.

The results of the segmentation were two sets of segmented geometries, one for the LV and one for the MV, each composed of 18 time frames representing one complete cardiac cycle. Thus, the positions of the structures of interest were known throughout one cardiac cycle. For contact management during systole, an artificial gap was imposed in the coaptation area of the valve, such that the anterior and posterior leaflets of the MV were not in complete closure during systole. In the following we will refer to the segmented geometries as “motion input meshes”, as they were used as input to prescribe the motion of the MV and LV during the simulations. The input meshes consist of triangulated surfaces, sharing the same topology (constant node numbers and triangles connectivity) during the cardiac cycle.

### Numerical model

The motion of the boundaries has been implemented with extensive own programing in a CFD solver which supports the ALE framework. In this case, all the CFD simulations were performed with the commercially available software Fluent (Ansys, release 15.0), which allows for the solution of the Navier–Stokes (NS) equations with a finite volume approach in an ALE formulation. For an arbitrary control volume V bounded by the surface A, the NS equations can be written in their integral form by incorporating the velocity of the computational grid $\vec{w}$ as:

1 $$\frac{\partial }{\partial t}\int_{V} {\rho_{f} dV} + \int_{A} {\rho_{f} \left( {\vec{v} - \vec{w}} \right) \cdot \vec{n}dA}$$

2 $$\frac{\partial }{\partial t}\int_{V} {\rho_{f} \vec{v}dV} - \int_{A} {\rho_{f} \vec{v}\left( {\vec{v} - \vec{w}} \right) \cdot \vec{n}dA} - \int_{A} {\sigma_{f} \vec{n}dA} = \int_{V} {\vec{f}_{f} dV}$$

where $\rho_{f}$ is the density of the fluid, $\vec{v}$ the fluid velocity and $\vec{n}$ the normal unit vector pointing out of the control volume. $\sigma_{f}$ the stress tensor and $\vec{f}_{f}$ the forces per unit of volume. An implicit, first-order accurate, backward difference method for the time discretization and a second order upwind scheme for the spatial discretization were used.

### Boundary conditions and material properties

As a starting point for the simulations, the late diastole was chosen, when the mitral valve was in the open position, the aortic valve was closed and the LV relaxed. The realization of the patient-specific model was performed starting from the available segmented rt-TEE images at this time-point (Fig. 1b). The motion input meshes of the MV and LV at late diastole were imported into pyFormex, an open-source, in-house written and python-based program which allows for the manipulation of large and complex geometries [http://www.nongnu.org/pyformex/]. The final fluid domain consisted of the left ventricle, the left ventricle outflow tract, a simplified atrium (not available from the images) and the mitral valve. The computational domain is shown in Fig. 1d.

The mesh of the computational domain was realized with about 500 K tetrahedral cells, resulting in an average element dimension of 1.2 mm. The dimension of the mesh was assessed with a mesh sensitivity study, reported in Additional file 1.

As boundary conditions, the motion of the LV and MV was mathematically described and implemented in the model with user-defined functions (more details in the following sections). All the remaining surfaces with the exception of the inlet surface are allowed to deform to follow the 3D complex motion of the annulus of the MV. The atrial surface was kept at a constant pressure throughout the entire cardiac cycle, as no pressure curves were available from the clinical data. The presence of the aortic valve is included with the on–off procedure, the switch between the open and closed configuration and vice versa was instantaneous and automatically prescribed based on the flow rate. During systole, the aortic outlet was kept at the constant pressure of 100 mmHg, while during diastole the surface was converted to a wall.

The blood was modeled as a homogeneous and Newtonian fluid, the values of density ρ and viscosity μ were chosen according to mean values for healthy subjects (${\rho = 1060\frac{\text{kg}}{{{\text{m}}^{ 2} }},\mu = 0.0035{\text{Pa}} \cdot {\text{s}}}$), thus neglecting the non-Newtonian properties of blood [20].

### Workflow

To prescribe the displacements of the structures of interest starting from the information available from the rt-TEE ultrasound imaging, three main steps were taken in the model. Prior to the simulation, a time interpolation was required to be able to transfer the displacement information from the time frames of the segmented meshes to the small time steps of the CFD simulations with smooth interpolating curves. As a second step, a space interpolation was required, to couple the motion of the input meshes and the computational mesh. As a last passage, before the solution of the NS equations, a contact detection algorithm was required, to ensure the correct treatment of the mitral leaflets coaptation during systole. These steps are described in details in the following sections and were implemented via user-defined functions, to modify the finite-volumes solving code according to the needs of the simulated scenario.

### Time interpolation

**Fig. 2:**
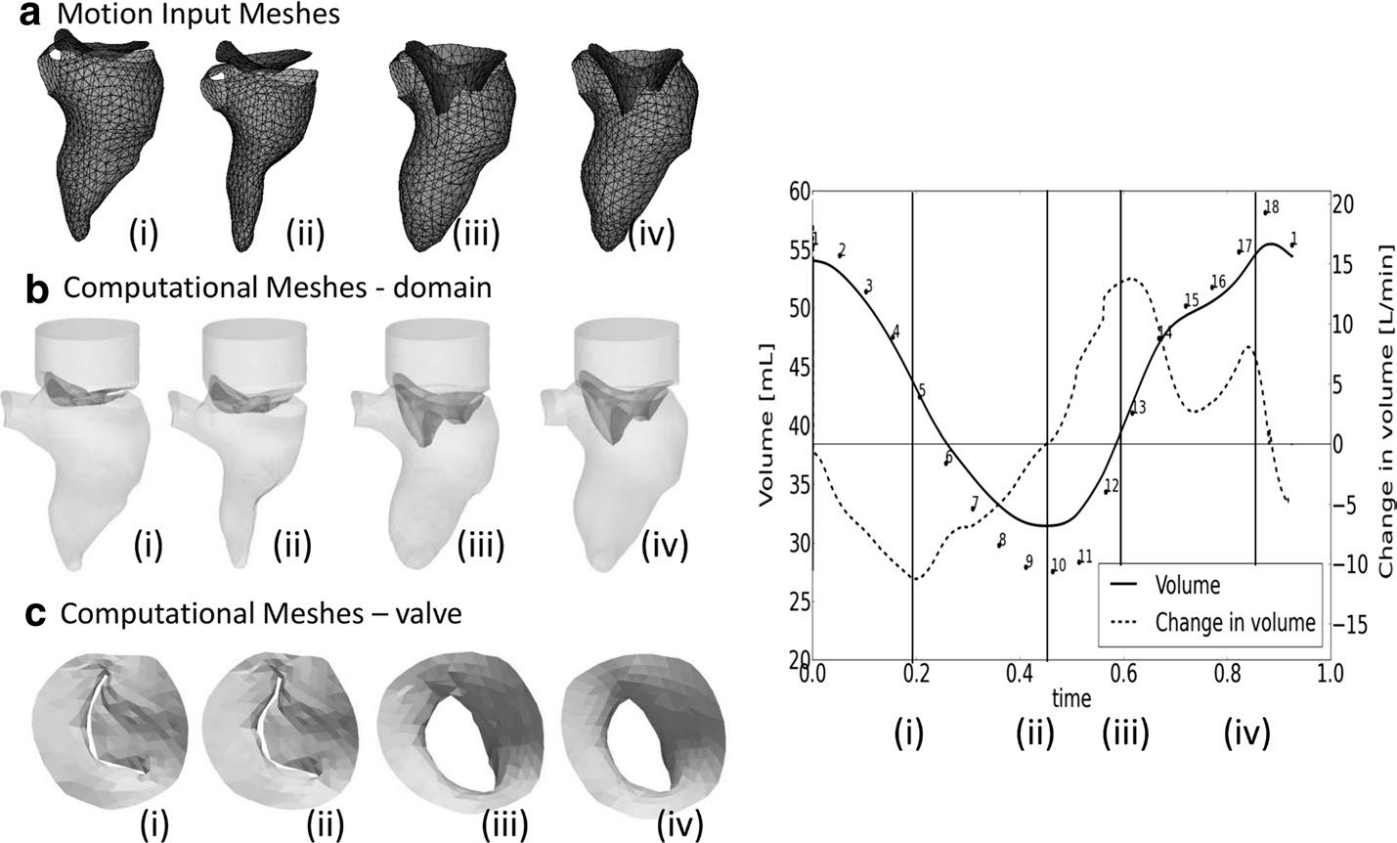
Prescribed motion of the boundaries. Comparison between (**a**) the motion input meshes and (**b**, **c**) the computed meshes, complete domain and *top view* of the MV, in four time-points of the cardiac cycle

The results of the segmentation process were two sets (one for the MV and one for the LV) of triangulated surfaces with a frame rate of 18 frames/cycle (Fig. 2a). This frame rate was not sufficient to replicate the motion of the walls in a CFD model (time-step used for the calculation equal to 1 ms), therefore a time interpolation was necessary. Natural Cubic Splines (NCS) and Bezier splines (BS) were selected to interpolate the displacements of the LV and MV respectively. The NCS were chosen for the LV as they ensure the continuity and smoothness of the curve and its derivative. The drawback of the choice of NCS over the BS is that they have less local control on the interpolated points: this could lead to some overshooting in the estimation of the interpolation curve, especially if the points are not widely spaced. For this reason, the motion of the valve was described with third order Bezier splines, which guarantees more local control on the interpolation despite the possible loss of continuity in the derivative. To account for the periodicity of the solution, the spline curves were calculated as closed curves, by appending the first frame after the last available frame. More considerations over this choice are presented in the discussion section.

### Space interpolation

With the imposed motion of the LV wall and valve, the position of the nodes of the fluid grid needed to be modified throughout the computation, which can be achieved in Fluent (as well as with other CFD packages) with a specific external function which updates the position of each point independently. Furthermore, it has to be taken into account that the segmented motion input mesh and the computational mesh have different resolutions. In the presented model, the updated position of the moving walls was obtained in three major substeps. As pre-processing step, the coefficients of the third-order interpolating curves are imported in the CFD solver. With reference to Fig. 1e, at each time-step, each boundary point x^i^ of the computational mesh is associated with the three closest points $\left( {{\text{x}}_{{0{\text{ref}}}}^{\text{i}} ,{\text{ x}}_{{1{\text{ref}}}}^{\text{i}} ,{\text{ x}}_{{2{\text{ref}}}}^{\text{i}} } \right)$ on the input surface. Knowing the spline coefficients, the position of each input point is updated. The projection $\left( {{\text{x}}_{\text{proj}}^{\text{i}} } \right)$ of each computational point on the triangle (T^i^) generated by the three nearest points on the input surface is calculated and its barycentric coordinates (α, β, γ) with respect to this triangle are calculated. Finally, the displacement of the computational point is calculated as a linear combination of the displacements of the three points weighted with the barycentric coordinates [21]. Further details about the space interpolation can be found in Additional file 2.

### Contact of mitral leaflets

Coaptation of the valve leaflets during systole is problematic for the ALE formulation as the complete closure of the MV would eventually result in the separation of the domain and consequently failure of the simulation. To overcome this limitation, prior the simulation a gap in the MV motion input meshes during systole was ensured. By doing so, the anterior and posterior leaflets never experienced a contact condition. Without any additional measures, this technique introduces an artificial transmitral backflow during systole. This was prevented with the development of a dedicated contact function which identifies the faces considered in contact and the fluid cells lying in the artificial gap. A triangular face and a node (which does not belong to an adjacent face) of the valvular surfaces are considered to be in contact if their distance is smaller than a predefined threshold based on the dimension of the artificial gap, if their normals are parallel (within a tolerance range) with opposite directions and if the center of one face projected along its normal falls within the second face. If these criteria are fulfilled, the face under consideration and the face to which the node belongs are considered to be in contact and all the fluid cells lying between them are then flagged for the addition of the momentum source, with an iterative identification process. Four iterations were sufficient to completely fill the gap. To increase the hydraulic resistance of the cells lying in the gap and therefore reduce the artificial backflow, an external momentum source term $\mathop \smallint \nolimits \vec{f}_{f}$ was introduced on the right-hand side of Eq. 2 for the identified cells. For each selected *j*th cell, this term resulted in:

3 $$f_{j} = C_{0} v_{j}$$

with *v*_*j*_ the fluid velocity in the *j*th cell and *C*_*0*_ a constant coefficient for the momentum source [22]. A separate set of tests was performed to identify a suitable value for the C_0_ coefficient (0.1 kg/s). This technique did not intend to mimic any physiological phenomenon and the constant coefficient of the momentum source term was chosen as a trade-off between the reduction of the mitral backflow and the stability of the solution procedure. Further details about the contact algorithm are provided in Additional file 2.

### Fluid-dynamics evaluation criteria

Besides the standard analysis of the flow field, of particular importance is the analysis of the vortex structure formation and evolution. The vortex structure can be identified with the λ_2_ criterion [23]. This method relates the vortex formation to the areas where minimum values of pressure are associated to the rotation of the flow field. It has been shown that by taking the symmetric and asymmetric parts $(S_{ij} S_{kj} + \varOmega_{ij} \varOmega_{kj} )$ of the velocity gradient tensor, the condition of the second invariant λ_2_ < 0 provides an indication for the vortex location [5, 23].

As local pressure differences in the ventricular chamber have an influence on the vortex formation and efficient ventricular filling, the pressure map at significant time-points is reported. Pressure values are relative to the uniform pressure assumed as boundary conditions. The intraventricular pressure difference ΔP is calculated between the section S_1_ near the base of the LV, and S_2_ near the apex (see Fig. 1d for the exact locations), with ΔP = pressure at base–pressure at apex. The wall shear stresses (WSS) are also reported.

As a proof of concept, in this paper we also report a comparison between the complete model and the model where the mitral valve was removed, to explicitly test and discuss the influence of the 3D valvular leaflets on the results of this type of simulation [9].

## Results

Two cardiac cycles have been simulated. The results from the second cycle (starting and concluding at late diastole, such that the influences of the closing valves are visible in the results) are presented in the following, to eliminate the influence of the initialization from the results. The simulations were run on a Dell PowerEdge R620 server (2 × Intel Xeon E5-2680v2 CPUs at 2.8 GHz) with one core used, and needed about 8 h to complete. The use of one core was required by the contact algorithm, which was not yet optimized for parallel calculation.

### Contact management

Without any increase in the hydraulic resistance of the fluid cells in the coaptation zone, the artificial gap between the leaflets in combination with the transvalvular pressure difference (100 mmHg) resulted in a significant backflow during systole (61 % of the total forward flow). The contact function, with the momentum source term in the NS equations and the selected *C*_*0*_ coefficient, reduced the unwanted backflow to 6 % of the forward flow. This residual transmitral flow during systole could not be, at this stage, further diminished, as additional increase of *C*_*0*_ led to numerical instabilities. The results shown in the following sections are obtained with the activation of the contact function.

### Motion of the boundaries

The described interpolation process, together with an optimal segmentation technique, generated a realistic boundary motion for the model, yielding physiological movement of the structures of interest. In Fig. 2, the comparison between rt-TEE ultrasound images, the segmented geometries (2a) and the corresponding time-points from the simulations (2b) are reported, at four instants throughout the cardiac cycle. In Fig. 2c the artificial gap is visible for the two time-points of diastole (Fig. 2c: i–ii).

The mass balance was fulfilled throughout the cardiac cycle, with the mass flow rate entirely defined by the motion of the structure, and the change in volume balanced by the inlet and outlet flows. The volume of the LV obtained from the motion input meshes was realistic. The described time interpolation did not introduce significant errors, as the maximum difference in the ejection fraction between the input geometries and the interpolated geometries is below 2 %.

### Flow field

**Fig. 3:**
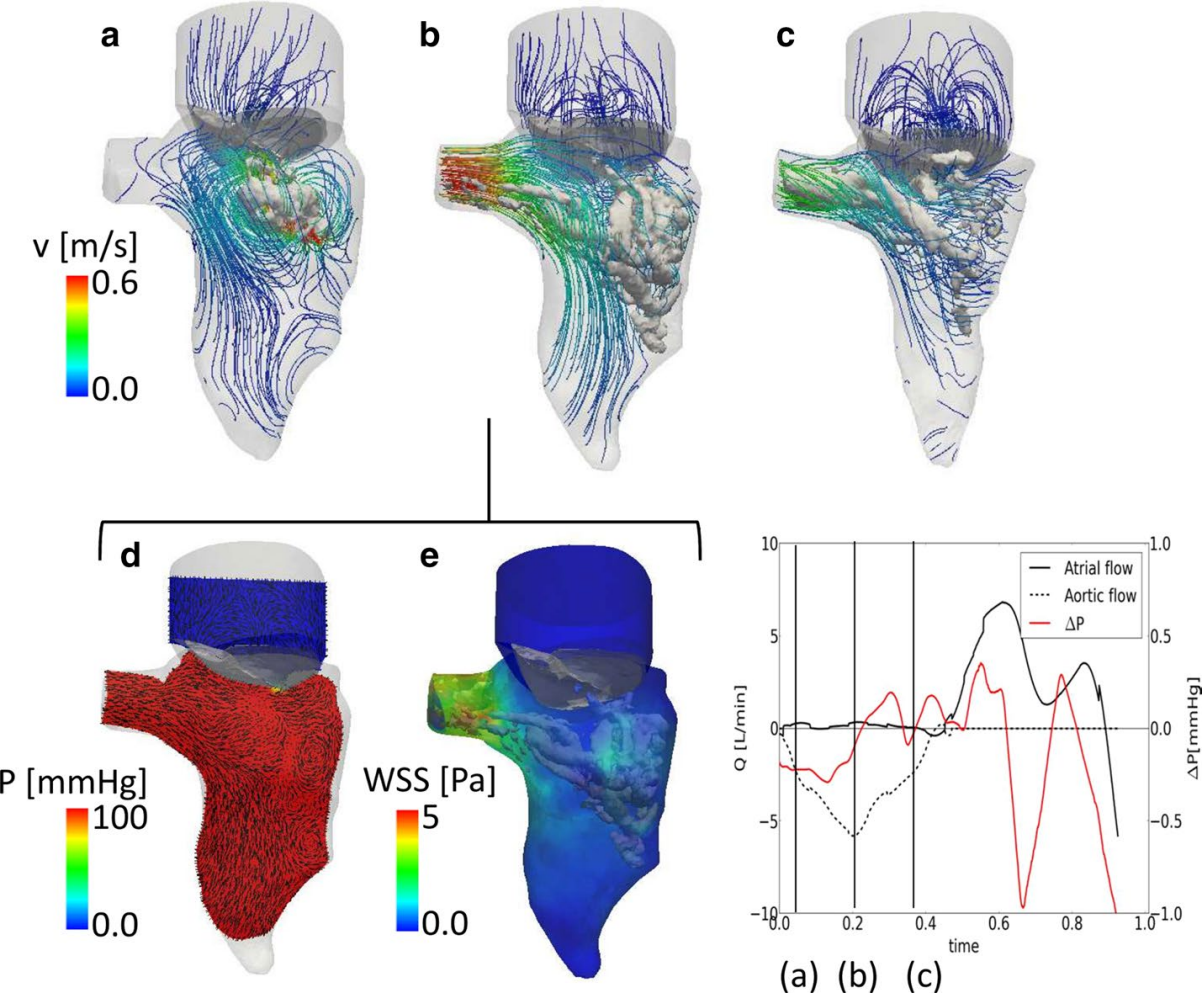
Systolic flow field. Streamlines and vortex structure (λ_2_) (**a–c**). Pressure and velocity vectors in the LV (**d**, **e**) on a section at peak of systole. **f** Flow curve and the intraventricular pressure difference (base–apex) during the cardiac cycle. The time-points used in **a–e** are indicated

The main flow field results at three significant time points in systole are reported in Fig. 3 (acceleration in Fig. 3a, peak of systole in Fig. 3b, deceleration in Fig. 3c) with an overlay of the deformation of the LV, the velocity streamlines and vortex structures. In Fig. 3d, the pressure field and the velocity vectors over a section at the peak of systole are reported, in Fig. 3e the WSS at peak of systole are shown.

At the beginning of systole (Fig. 3a), a limited flow is present at the aortic outlet, as the MV ended its closing phase and contraction of the left ventricle has yet to occur. A low intensity recirculation area is visible in the center of the LV, in the area below the valvular orifice (Fig. 3a). Due to the periodicity of the solution, this vortex is a residual of the vortex generated during the preceding diastole. The closure of the mitral valve could also contribute to the formation of this structure and could especially influence its location, close to the lateral wall of the LV. As soon as the contraction of the LV begins, the flow vectors align, culminating at the peak of systole (Fig. 3b), when the velocity of the blood leaving the LV is the highest (v_max_ = 0.6 m/s) and the reduction of the volume of the LV is already significant. At this stage, a complex vortex structure is delineated at the lateral wall of the LV (Fig. 3b). The blood decelerates during the late systole, while the LV concludes its contraction. In Fig. 3c, the LV reaches its minimal volume at the end of the systolic phase, and the deceleration of blood gives rise to a helical flow pattern at the aortic location. The vortex structures are washed out of the ventricle during the late systolic phase. During the entire systolic phase, the atrio-ventricular pressure difference is about 100 mmHg, coherent with the imposed boundary conditions (Fig. 3d). The contact implementation guarantees a good sealing of the valve despite the presence of the artificial gap. Smaller intraventricular pressure differences are not visible in the color plot of this cardiac phase, as they are of two/three orders of magnitude smaller than the transvalvular pressure difference. As visible in the graph (Fig. 3f), however, the intraventricular pressure (calculated as the average pressure difference between the surface S1 at the base and the surface S2 at the apex of the LV (Fig. 1d) shows small pressure variations (±0.2 mmHg) in the ventricle. In the acceleration phase (until t = 0.25 s), there is an expected negative intraventricular pressure, while during the deceleration phase the intraventricular pressure difference is inverted due to the blood deceleration. In Fig. 3e, the wall shear stress plot at peak of systole shows high values at the aortic outlet, as the velocity of the blood in this region is the highest. Areas of relatively high WSS are also detected in the region where the vortex structures are present.

**Fig. 4:**
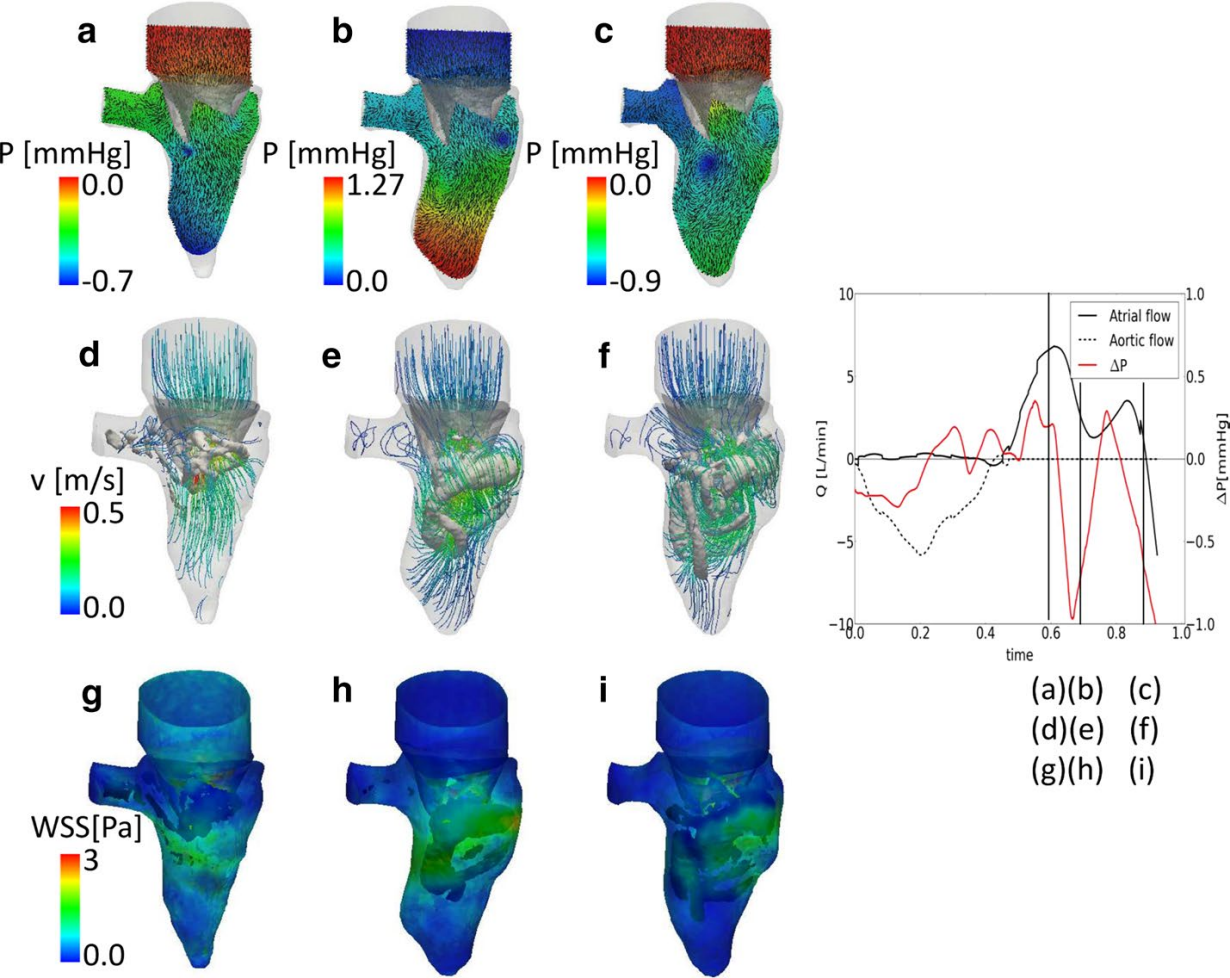
Diastolic flow field. Pressure and velocity vectors in the LV (**a–c**) on a section. Streamlines and vortex structure (λ_2_) (**d–f**). WSS at the walls (**g–i**). **j** Flow curve and the intraventricular pressure difference (base–apex) during the cardiac cycle. The time-points used in **a–i** are indicated. Please note that the *color scale* for the pressure difference changes in each panel

The results during diastole are shown in Fig. 4. Figure 4a–c reports the pressure and the velocity vectors on a section, Fig. 4d–f the velocity streamlines and vortex structures, Fig. 4g–i the wall shear stress. During the diastolic phase, the pressure in the ventricle decreases to the atrial pressure, in this case 0 mmHg, as imposed from the boundary conditions. At early diastole (Fig. 4a), a negative intraventricular pressure is generated by the blood entering the ventricle (E-peak). The intraventricular pressure is then reversed during the blood deceleration phase (Fig. 4b) to become again negative during the A-peak towards the end of the diastole (Fig. 4c). The variation of the intraventricular pressure difference (base–apex) throughout the cardiac cycle is reported in Fig. 4j. Please note that during diastole, the intraventricular pressure difference is expected to be positive during acceleration and negative during the deceleration phase, due to the location of the planes on which the pressure difference is measured. This is the opposite to what is expected in systole, due to the opposite direction of the blood flow.

The early diastolic flow (E-peak) is characterized by a peak velocity in the valvular orifice area. A vortex structure is generated at the valvular edges, visible both from the small negative pressure areas at the leaflets in Fig. 4a and from the vortex and streamlines in Fig. 4d. During the deceleration phase, the vortex has significantly increased in size and detached from the valvular edges (Fig. 4e). The shape of the vortex becomes complex and it migrates towards lower regions of the LV. At the lateral wall, the vortex impinges the ventricular wall (Fig. 4e), starting the dissipation [24]. The dissipation of the vortex continues during the late diastolic phase, as visible in Fig. 4f. The second peak (A-peak) is not clearly visible from Fig. 4f, as the flow field is chaotic and the vortex structure widespread in the LV. The A-peak is detected in the ventricular motion and in the transmitral flow, but no second re-opening phase of the mitral valve is detected. The WSS is higher in the regions where the vortex reaches the ventricular walls. The mitral leaflets are also subjected to high values of WSS, due to the transmitral flow during diastole.

For a global overview on the flow field results, a movie is provided as Additional file 3.

### Influence of the valvular leaflets

**Fig. 5:**
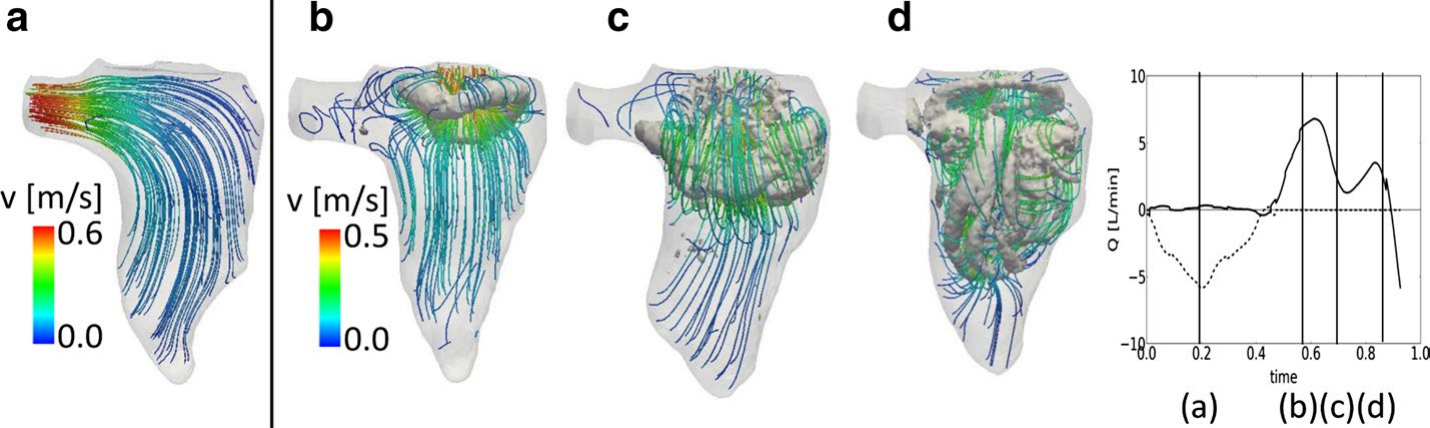
Flow field for the model without the valve: vortex and velocity streamlines at peak systole (**a**) and in three time-points during diastole (**b–d**)

As the presence of the valve significantly influences the flow field and the vortex formation in the LV [9], we also compared the same model with and without the MV. An additional model was created with an analogue geometry in which the valve was removed and replaced with an orifice. The orifice was obtained by projecting the widest MV aperture on the valvular plane, to ensure a peak velocity comparable to the velocity measured in the model with the full 3D valve. The aperture of the valve orifice was considered constant in time and the valve operated in an on/off mode as described for the AV. In Fig. 5, the results of the model without the valve are shown at peak systole (Fig. 5a) and in diastole (Fig. 5b–d).

At peak systole (Fig. 5a), the flow is nicely aligned with the outlet and no vortex structures are detected at this point, while in Fig. 3b some disturbance of the flow is present and residual vortex structures have accumulated near the lateral wall of the LV. At the beginning of diastole (Fig. 5b), the vortex arising from the valvular orifice is much more regular than the vortex that is obtained with the 3D valve. The vortex ring has the saddle shape of the orifice, but the absence of the leaflets prevents the higher velocities at the commissures and therefore the generation of an irregular and curved vortex structure. In Fig. 4c, d the difference becomes more clear. In the model with the valve, the vortex is pushed by the valvular leaflets towards the lateral and septal walls, leading to the dissipation of the structure. Without the valve, the vortex remains in the central zone of the LV, travelling forward towards the apex without significant dissipation (Fig. 5c). With the valve, the flow streamlines are more complex and the fluid structures reach more apical zones of the left ventricle. Without the valve, less blood reaches the apex, leading to a less optimal washout of the area. These findings are in good agreement with [9].

## Discussion

In this paper, we have described a patient-specific CFD model of the left ventricle, including the moving ventricular wall and the full three-dimensional mitral valve segmented from rt-TEE images. The methodology offers advantages over the existing works, in particular for (i) the use of clinically available rt-TEE ultrasound images as a base for the motion, (ii) the inclusion of the full 3D valve in the model and (iii) the use of the ALE technique for the discretization of the NS equation. In particular, the availability of the ultrasound images, routinely acquired during any surgical procedure, cheaper and less invasive for the patient, provide a good ground for the generation of a model with the potential to become a clinical evaluation and/or surgical planning tool. Previous works based on ultrasound have been proposed [24, 25], but the valvular structure was not included in their model.

### Rt-TEE images

While the real-time TEE echo data offers the advantage of a sufficient spatial resolution to resolve and segment the mitral valve apparatus and leaflets, the frame rate is relatively low, which does not allow to include the iso-volumetric phases of the cardiac cycle and enforces the use of temporal interpolation. As also, the opening and closing time of the valve were determined by the frame rate of the reference meshes, the model does not provide any insights into the opening and closing time of the MV. However, the frame rate of the rt-TEE images was sufficient to capture the significant events of the cardiac cycle, for example the typical two peaks of the transmitral diastolic flow and the transmitral velocity magnitudes which are realistic.

### Modeling choices

The use of NCS to interpolate the reference points of the ventricular mesh overcame some of the limitations of the available studies [8, 10–12]. The choice of NCS for the interpolation of the displacement of the LV resulted in a wall motion and ejected volumes which were coherent and physiologically plausible (Fig. 2), yielding more realistic simulation results than previously used interpolation schemes [8, 10–12]. Nevertheless, the choice of using BS to describe the motion of the leaflets was visible in the flow curve (Fig. 3) where small discontinuities were visible. However, as the bulk flow is defined by the LV motion, and therefore prescribed with a smooth and continuous curve, we expect that this has only a minor effect on the overall fluid dynamics.

The use of the ALE technique for the NS equations requires specific attention for the treatment of distorted cells that inevitably occur in a deforming geometry such as a LV. The chosen approach, with an adaptive update of the reference and the computational meshes, removed the need for intermediate meshes, as previously used in intraventricular flow studies based on the ALE method [8, 10–12]. The drawback of such approach is the need of remeshing in the fluid domain, which increases the computational time and requires the use of a tetrahedral grid in the chosen software package.

### Intraventricular flow

Given that the presented model is based on imaging data from a patient with aortic stenosis, the results obtained reflect the patho-physiology of the case under investigation. The relatively low magnitude of the volume and flow curves is related to the ventricular pathology of the patient and the small size of the heart, confirmed by the clinical intra-operative report. The capability of the model to capture interesting details of the intraventricular haemodynamics was shown in Figs. 3 and 4. In particular, the CFD modeling allowed for the analysis of variables which are otherwise not available from the clinical images, e.g. 3D vortex visualization. The structure and the evolution of the vortex structure throughout the cardiac cycle were in agreement with the published data for a similar set up [5, 7, 9, 16, 26]. The patient-specific details of the valvular anatomy played an important role in the vortex formation and the asymmetry of the mitral valve resulted in a saddle-shaped vortex ring [7, 9, 16]. The transvalvular pressure difference during systole is coherent with the imposed boundary conditions, showing the capability of the contact implementation of sealing the valve during this phase. The model also yields physiologically plausible intraventricular pressure variations, which may be of clinical interest especially for the assessment of diastolic function [5]. The analysis of the WSS at the ventricular walls showed the presence of high values of WSS close to the vortex location during the cardiac cycle. In fact, the blood velocities generated at the vortex core become tangential to the walls when the vortex structure approaches it, therefore resulting in areas of high WSS values [24].

The comparison between the model with and without the valvular leaflets (Fig. 5) highlights the importance of the presence of the valve in the computational model to achieve realistic features of the vortex field, in particular the penetration of the vortex ring to the apical region. These results are in line with observations of Schenkel et al. comparing CFD model results with MRI flow recordings and with the findings of Seo et al. who studied the impact of the presence of a valve in a CFD model based on LV CT scans and a physiology-inspired model valve.

### Limitations and future work

Previous studies have demonstrated that the upstream boundary conditions can have an impact on the ventricular flows [7, 12]. In our model, the geometry of the atrium was very simplified, as the field of view of the rt-TEE images was limited to the LV and MV. Furthermore, probably due to the low frame rate, the mitral leaflets do not experience an appreciable second opening during the A-peak of diastole. More realistic upstream geometries will be taken into account in the future. The aortic valve and the inner topology of the LV (trabeculae and papillary muscles) were not included in the model yet. In the presented model, the torsion of the ventricle could not be directly analyzed. Due to the interpolation process from the input mesh to the computational mesh, the in-plane motion is not represented accurately. Consequently, no torsional effects of the ventricle can be discussed. The assumption of the Newtonian model for the blood also introduces approximations in the results, as the blood in the ventricle, especially in the apex, can reach very low velocities, at which the Newtonian hypothesis is no longer fully valid [12]. More sophisticated models for the blood will be investigated in the future.

There are a number of additional hypothesis which can also affect the accuracy of the results, among others, the presence/absence of the papillary muscles [16, 27] or the presence of beat-to-beat differences and fluctuations due to high frequency components of the flow [27].

The model was tested over only one data-set as a proof of concept and a further comparison with ultrasound Doppler or 4D MRI data has to be performed for validation.

## Conclusions

In this paper we have described a methodology that allows to construct a patient-specific CFD model with imposed moving boundaries, including the three-dimensional mitral valve obtained from real-time transesophageal ultrasound images. The model uses uniquely the ALE framework and it is the first time a realistic valve has been included with this approach. Besides the patient-specific and anatomically correct nature of model, the presented interpolation and remeshing methodology is numerically efficient, and compliant with an ALE formulation thanks to the introduction of a leaflet contact detection algorithm. The model provides detailed intraventricular flow features, and highlights the importance of the 3D valve apparatus for the vortex dynamics and apical flow. The methodology might therefore be of particular importance in patient treatment planning to assess the impact of mitral valve treatment on intraventricular flow dynamics.

## Additional files

10.1186/s12938-016-0231-9 Report of the sensitivity test analysis.
10.1186/s12938-016-0231-9 Pseudo-code version for technical details on the moving boundary and contact algorithms.
10.1186/s12938-016-0231-9 Flow field results during the cardiac cycle.

## Abbreviations

CFD: computational fluid dynamics
3D: three dimensional
LV: left ventricle
MV: mitral valve
MRI: magnetic resonance imaging
CT: computed tomography
ALE: arbitrary Lagrangian–Eulerian
IB: immersed boundary
rt-TEE: real time trans-esophageal
NS: Navier–Stokes
NCS: natural cubic spline
BS: Bezier spline
