# Supplementary material for: Patient-specific CFD simulation of intraventricular haemodynamics based on 3D ultrasound imaging
Source: Biomed Eng Online. 2016 Sep 9;15(1):107. doi: 10.1186/s12938-016-0231-9 (PMC5016944; doi:10.1186/s12938-016-0231-9)
Supplement: Supplementary file 1 — 10.1186/s12938-016-0231-9 Report of the sensitivity test analysis. [file 12938_2016_231_MOESM1_ESM.pdf]

Appendix 1: grid sensitivity analysis

A sensitivity analysis was performed, to identify the most suitable dimensions for the computational mesh. Four different meshes were generated (from 50K to 500K cells) and the dimensions are reported in table (a). The mesh of 50K is the same used in the first version of the manuscript. An identical simulation was performed for the 4 cases, coherent with the parameters and working conditions described in the Materials and Methods section of the manuscript. The computing time for each case is reported in table (1). The time-step size was chosen for each simulation such that the mesh motion was always leading to a valid mesh (no inverted elements). The chosen time-step size was 1ms for the 50K, 100K and 160K cells, while a time-step size of 0.5ms was adopted for the 500K cells simulation.

Table A1: grid dimension and computing time

Average element number	Computing time
**50K**	5h
**100K**	8h
**160K**	12h
**500K**	5d

Various performance indexes were evaluated and are reported in figure a. First, integral quantities over the entire domain were calculated. We report the average velocity (fig A1.a), the vorticity (fig A1.b) and the (viscous) energy dissipation (fig A1.c). The latter quantity is derived from the NS equation and the conservation of energy combined with the assumption of an homogeneous, incompressible, Newtonian and isotropic fluid as:

$$\Phi = 2\mu\left[\left(\frac{\partial u}{\partial y}\right)^2 + \left(\frac{\partial v}{\partial y}\right)^2 + \left(\frac{\partial w}{\partial z}\right)^2\right] + \left(\frac{\partial u}{\partial y} + \frac{\partial v}{\partial x}\right)^2 + \left(\frac{\partial u}{\partial z} + \frac{\partial w}{\partial x}\right)^2 + \left(\frac{\partial v}{\partial z} + \frac{\partial w}{\partial y}\right)^2$$

As a clinically relevant quantity, we report the intraventricular pressure difference (base – apex, surfaces location indicated as S1 and S2 in figure 1) during the entire cycle (figure A1.d). As high values of wall shear stress could be indicators of damage on the ventricular walls or valvular leaflets, in figure A1.e we report the surface area of the LV and MV subjected to high shear values (calculated as the area with a WSS value higher than the 80% of the peak shear stress on the coarsest mesh). In figure A1.f the surface area of the vortex structures (identified in figure 3 and 4) is reported for the four cases.

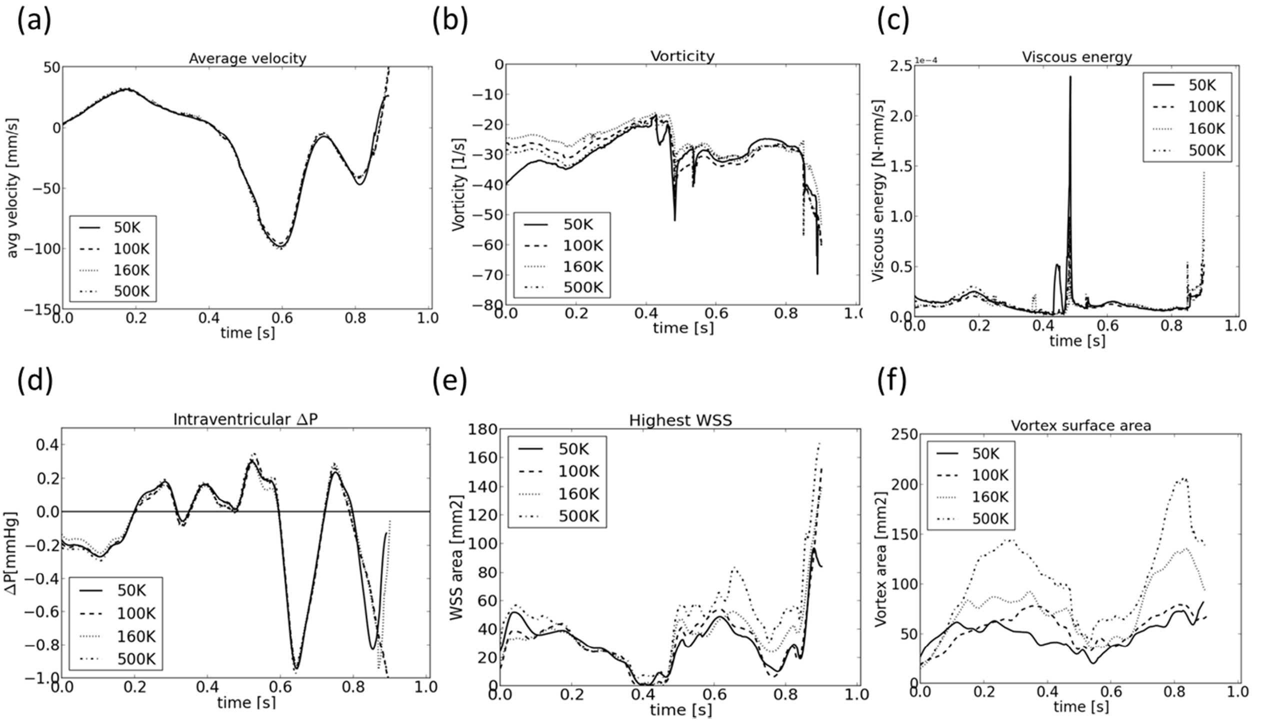

Figure A1: (a) average velocity, (b) vorticity, (c) viscous energy, (d) intraventricular pressure difference, (e) surface area of highest WSS values, (f) surface area of vortex structures in the four different meshes.

From figure A1(a-d) no significant differences are visible in the global features of the flow field. The high peak visible in the viscous energy plot is due to the sudden closure of the aortic valve.

The most significant differences are visible in figure A1.f, in which the surface of the vortex significantly increases with the degree of refinement of the mesh. Having a finer mesh, the vortex structure and vortex breakup is more represented and smaller features are captured by the simulations. Consistently, a larger region of high WSS is detected in more refined meshes especially during diastole, as the area close to the walls occupied by the vortex is larger (figure A1.e). As a visual example, we report the differences in the vortex structure in figure A2 during the deceleration phase of diastole (t=0.65s), at which the vortex structure is the largest.

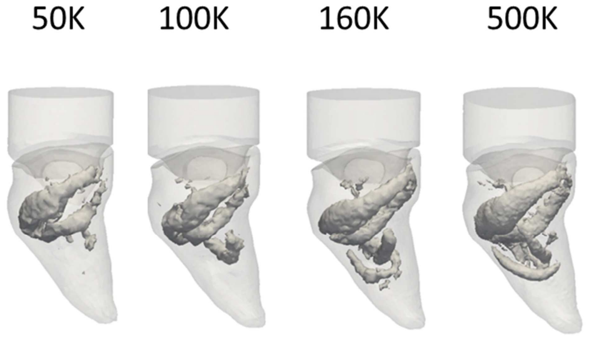

Figure A2: vortex structures in the 4 analyzed cases (t=0.65s).

All grids capture the main vortex structure during diastole. However, only the more refined meshes can capture the secondary structures of the flow. For this work, the 500K cells mesh was selected. Given the small dimensions of the ventricle under investigation, for the 500K cells grid the average cell size is of 1.2 mm. The obtained grid is not uniform in space, as a more refined mesh is required in the neighborhood of the valve. The dimension of the cells (especially in the valvular region) is comparable to the size reported in Chnafa et al., 2014 (0.8mm average value for an almost uniform grid) for the entire and physiological left heart including the atrium and the vessels.
