# Supplementary material for: Patient-specific CFD simulation of intraventricular haemodynamics based on 3D ultrasound imaging
Source: Biomed Eng Online. 2016 Sep 9;15(1):107. doi: 10.1186/s12938-016-0231-9 (PMC5016944; doi:10.1186/s12938-016-0231-9)
Supplement: Supplementary file 2 — 10.1186/s12938-016-0231-9 Pseudo-code version for technical details on the moving boundary and contact algorithms. [file 12938_2016_231_MOESM2_ESM.pdf]

Appendix 2: Pseudo-code for UDF.c

Initialization:

```
- loop over the input surfaces
        - loop over the input points
                - read the spline coefficients for the position of this input point as calculated
                in pyFormex
```

Space Interpolation (find the nearest points):

```
- loop over the moving computational surfaces
        - loop over the faces
                - loop over the nodes
                          - calculate the distance with all the points (at time=t) of the
                          corresponding input surface
                          - identify the 5 nearest points of the corresponding input surface
                          - store the IDs of these closest input points
```

Update of the Fluent Mesh:

```
- loop over the input surfaces
        - loop over the points
                - update the position of this point using the spline coefficients stored in Fluent
                - calculate the displacement with respect to the previous time step
- loop over the moving computational surfaces
        - loop over the faces
                - loop over the nodes
                          - retrieve the 5 nearest input points
                          - check the quality of the triangle formed by the nearest input points
                          (select the 3 closest points which generate a not-skewed triangle)
                          - project the node on this input triangle
                          - calculate barycentric coordinates of projection
                          - if (projection is outside the triangle) then force the projection of
                          the Fluent point on the closest edge or point of the input triangle
                          - update the position of the Fluent point as linear combination of the
                          displacements of the 3 closest input points
```

Contact detection:

```
- set thresholds for contact detection
- loop over all the faces (f1) which can possibly be in contact
        - calculate the face normal
        - loop over the nodes (n1)
                - check distance of node (n1) from center of face (f1)
                - if distance < threshold
                               - calculate node normal
                               - check direction of face and node normal
                               - if (normals almost parallel and with opposite sense)
                                       - if the projection of the node along its normal falls
                                       within the original face then set flag for contact on the
                                       face (f1), the node (n1) and on the faces (f2) to whom the
                                       node n1 also belongs
```

Fill the gap:

```
- loop over the moving computational surfaces of valve
        - loop over the faces
                - retrieve the flags for contact
                - loop over the cells neighboring the flagged faces
                          - set contact-flag in this cell
                          - loop over the cells sharing one face with this contact-flagged cell
                          until a wall is found or a max number of iterations is reached
                               - set contact-flag in this cell
```
